# Fungal Septic Knee Arthritis Caused by *Aspergillus fumigatus* following Anterior Cruciate Ligament Reconstruction

**DOI:** 10.3390/diagnostics11111975

**Published:** 2021-10-24

**Authors:** George Samonis, Christos Koutserimpas, Georgia Vrioni, Elsa Kampos Martinez, Panagiotis Kouloumentas, Kalliopi Alpantaki, George Saroglou

**Affiliations:** 1 Department of Internal Medicine, University Hospital of Heraklion, Crete, 71500 Heraklion, Greece; 2 “Metropolitan” General Hospital, N. Faliron, 18547 Athens, Greece; espiro517@yahoo.com (E.K.M.); info@kouloumentas.gr (P.K.); gs200744@otenet.gr (G.S.); 3 Department of Orthopaedics and Traumatology, “251” Hellenic Air Force General Hospital of Athens, 11525 Athens, Greece; chrisku91@hotmail.com; 4 Department of Microbiology, Medical School, National and Kapodistrian University of Athens, 11527 Athens, Greece; gvrioni@med.uoa.gr; 5 Department of Orthopaedics and Traumatology, “Venizeleion” General Hospital of Heraklion, Crete, 71409 Heraklion, Greece; apopaki@yahoo.gr

**Keywords:** *Aspergillus* arthritis, fungal arthritis, knee infection, anterior cruciate ligament infection

## Abstract

Postoperative infections after arthroscopic anterior cruciate ligament (ACL) reconstruction (ACLR) represent a rare but severe complication. An extremely rare case of *Aspergillus* septic arthritis in a 27-year-old patient following arthroscopic ACLR is reported. The patient presented with signs of knee infection 14 days after ACLR. Two consecutive arthroscopic debridements were performed, while eventually histopathology, cultures and multiplex PCR test revealed *Aspergillus* identified as *A. fumigatus* by mass spectrometry. The patient commenced long-term treatment with voriconazole. Fungal arthritis or osteomyelitis following ACLR has a mild local and general inflammatory reaction when compared to the bacterial ones. Nevertheless, such infections may lead to aggressive osseous destruction and necrosis. A high index of suspicion is of utmost importance for early detection, while microscopic, histological examination and multiplex PCR may be more helpful for the diagnosis than cultures since cultures are more time-consuming and may vary depending on different factors.

## 1. Introduction

Postoperative infections following arthroscopic anterior cruciate ligament (ACL) reconstruction (ACLR) represent a relatively rare complication, ranging from 0.3% to 1.7% [1]. In the majority of cases, this complication does not lead to catastrophic outcomes [1,2]. Nevertheless, the treatment may be extremely challenging.

Coagulase-negative *Staphylococci* represent the most common organism in such cases, while fungal infections are extremely rare, with only a few reports exist so far in the literature [2,3].

An extremely rare case of fungal septic arthritis caused by *Aspergillus fumigatus* in a 27-year-old healthy patient following arthroscopic ACLR is presented. Additionally, a thorough review of the literature is provided.

## 2. Case Presentation

A 27-year-old male had an isolated ACL injury of the right knee due to a football accident. His medical history was unremarkable and he was not receiving medication for any reason. Ten days following the initial injury, the patient underwent, under general anesthesia, arthroscopic ACLR with hamstring tendon autograft. Tibial fixation of the graft was performed with the use of a bioabsorbable interference screw, while femoral fixation was performed with a button. The patient received perioperative antimicrobial prophylaxis with intravenous (iv) cefuroxime, while the harvested autograft was soaked in 1 g of vancomycin prior to insertion. The surgery lasted 70 min.

**Figure 1:**
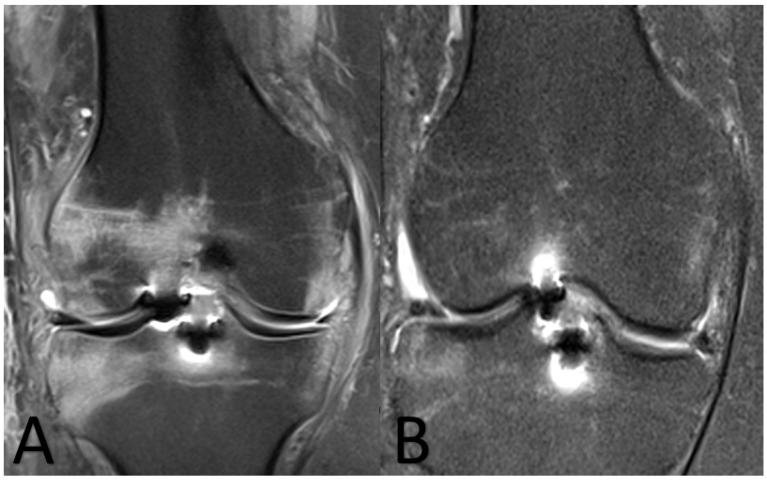
Coronal MRI views of the knee. (**A**) Fourteen days after the initial anterior cruciate ligament reconstruction; diffusely increased T2 signal/bone marrow edema is noted surrounding the ligament anchors, extending to the lateral femoral condyle, lateral tibial plateau, intercondylar eminences and the intercondylar notch. (**B**) Six months after antifungal treatment; imaging findings have notably subsided, indicating response to treatment.

Fourteen days following the procedure, the patient presented with fever (38.2 °C) and fatigue, while the surgically treated knee became swollen and warm. Laboratory findings revealed C-reactive protein (CRP) = 19 mg/L and erythrocyte sedimentation rate (ESR) = 51 mm/h, while magnetic resonance imaging (MRI) of the knee revealed signs of septic arthritis (Figure 1A). At that point in time, a decision for reoperation was made. He underwent knee arthroscopy. Cultures of the synovial fluid were obtained, while joint drainage and lavage with 10 L of normal saline were performed. No pus was drained from the knee joint. Intraoperatively, mild synovial swelling and hyperemia were observed, while the cartilage and the graft were not macroscopically altered. The cultures did not yield any pathogens. The patient was discharged on oral empirical antimicrobial treatment including ciprofloxacin and clindamycin.

**Figure 2:**
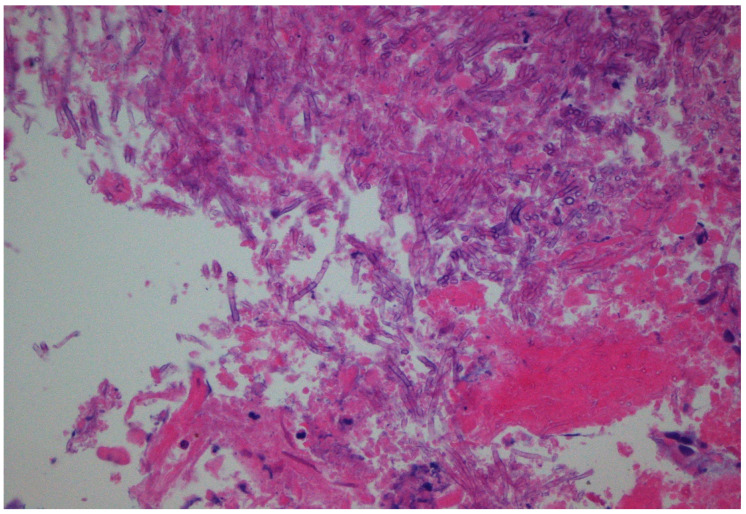
Histopathology of the removed graft revealed regularly septate hyphae branching at 45° angles (hematoxylin and eosin stain, magnification ×40).

On the 33rd postoperative day from the initial ACLR, the patient presented with deteriorating symptomatology, including pain with tenderness around the affected knee and prolonged fever (38.5 °C). The laboratory findings included CRP = 33 mg/L and ESR = 101 mm/h. At that point, he underwent additional arthroscopic debridement. This time, turbid yellow fluid was drained, while meticulous irrigation and debridement were performed. The autograft was removed and sent along with the drained fluid for microscopy, cultures and multiplex PCR and additionally sent for histological examination (Figure 2). Overdrilling of the femoral and tibial tunnels was also performed.

Microscopic examination of the autograft with 20% potassium hydroxide (KOH) revealed hyaline, septate, acute-angle branching hyphae. The autograft cultures on the Sabouraud dextrose agar (SDA) with 100 mg/L chloramphenicol after incubation in 30 °C yielded within 48 h velutinous grey–blue–green colonies. The colony from the SDA Petri dish was identified using (i) direct microscopy and (ii) the matrix-assisted laser desorption/ionization–time-of-flight (MALDI–TOF) mass spectrometry (MS) technology (Bruker Biotyper, Bruker Daltonik GmbH, Leipzig, Daltonics, Germany) by ethanol/formic acid (EtOH/FA) extraction and using the Bruker Biotyper library database (BDAL filamentous fungi/577 MSPs). Microscopy of the cultured organisms as well as identification with a Bruker Biotyper revealed *Aspergillus fumigatus* (score with Bruker Biotyper > 2.00). Moreover, the multiplex PCR of the synovial fluid for *Aspergillus* spp. (a genesig Standard RT-PCR detection kit for *Aspergillus*, PrimerDesign, UK), was positive for *Aspergillus* spp. Antifungal susceptibility testing was performed using the MIC method (EUCAST standardized broth microdilution method) (amphotericin B: 0.5 mg/L (S), isavuconazole: 0.025 mg/L (S), itraconazole: 0.006 mg/L (S), posaconazole: 0.025 mg/L (S), voriconazole: 0.006 mg/L (S)).

The patient then commenced (iv) voriconazole 2 × 400 mg on the first day and then, from the second day, 1 × 300 mg. Thirty-six hours following the autograft removal and the initiation of the antifungal treatment, the patient became afebrile, while the topical symptomatology (warmth and swelling) of the affected knee were significantly recessed.

The patient was discharged 14 days following last arthroscopy on oral voriconazole (300 mg) and linezolid (600 mg × 2). Linezolid was discontinued after 10 days, while the laboratory findings at that point revealed CRP = 0.6 mg/L and ESR = 19 mm/h. The patient at the 16-month follow-up did not have any signs or symptoms of infection, while per os voriconazole treatment continued for 6 months after the fungal diagnosis. At that point in time, new MRI of the knee revealed disappearance of the infection (Figure 1B).

## 3. Discussion

Infection following ACLR represents a rare but challenging complication. Its incidence is estimated to be between 0.14% and 1.7%, with *Staphylococcus aureus*, *Staphylococcus epidermitis* and *S. haemolyticus* being mainly blamed as causative pathogens [1]. Such infections should not be taken lightly since they may lead to devastating results, such as chondrolysis, osteomyelitis and osteochondral destruction, all of which may drastically alter the quality of life of a generally healthy young patient. Early and prompt treatment of such infections has been proven successful in many cases, even with ACL graft retention [1,4].

Fungal infections following ACLR are extremely rare, with only 14 cases described so far in the literature, with only two *Aspergillus* cases [2,3,4,5,6,7,8,9] (Table 1).

Fungal infections, in contrast to bacterial ones, lead to fewer tissue reactions and have a more indolent presentation [2,10]. In the reported case, the macroscopic image of the ACL graft during the first arthroscopy remained unaltered, without signs of infection. Hence, it was decided to retain the autograft.

In deep-tissue fungal infections, such as joint and osseous ones, the status of the host’s immune system seems to play an important role since these infections have been associated with immunocompromised patients [11,12,13]. It is of note that the reported patient was immunocompetent, with unremarkable medical history. Furthermore, in these rare cases of fungal arthritis following ACLR, it seems that most patients are relatively young, without immunosuppression or comorbidities. Fungal infection due to intraoperative contamination should be considered, especially in such a case without any risk factors [9]. However, no other such infection has been recorded in the same institution for a prolonged period. Additionally, it must be noted that the operating theaters, as well as the sterilization department are routinely decontaminated. Given all these factors, although an intraoperative contamination could not be excluded, in this case, it seemed extremely unlikely.

In this case, for the diagnosis, traditional and non-traditional diagnostic procedures were used. Moreover, for the identification of the fungi that grew in the culture dish, microscopy and mass spectrometry (Bruker Biotyper) were used. According to the revised EORTC/MSG criteria for defining invasive fungal infections, including invasive aspergillosis, a microbiological and/or histopathologic diagnosis is required for a proven infection [14].

*Aspergillus* species are found worldwide in soil and decaying matter. Invasive *Aspergillus* infections are typically seen in patients with significant underlying immunosuppression [15,16]. The clinical importance of *Aspergillus* spp. infection has increased as the number of immunocompromised patients has risen during the last decades [15]. Antifungals recommended for treatment of patients with invasive aspergillosis include voriconazole and amphotericin B, with cidal activity and echinocandins, that have only static activity against this mold. Patients with such infections often require prolonged antifungal treatment [17,18].

Voriconazole, that was introduced in 2003, has been proven the drug of choice against *Aspergillus* spp. [19]. This agent has changed dramatically *Aspergillus* spp. infection management. This agent, having all the characteristics of azole compounds, is moderately hepatotoxic and much less nephrotoxic than all amphotericin compounds [18]. The reported patient commenced voriconazole, while antifungal treatment lasted 6 months. The patient tolerated the prolonged treatment well, without any side effects or complications.

An increased rate of resistance to azole compounds has been reported in the Netherlands and the United Kingdom due to the extensive use of these agents as pesticides. The prevalence of azole resistance reportedly remains low in other countries [17]. However, this phenomenon may predict a future threat for other countries as well. Thus, it is of paramount importance to perform susceptibility testing to obtain accurate MIC values following *Aspergillus* spp. isolation. Nevertheless, it must be noted that for a number of molds, including *Aspergillus*, laboratory methods indicating MICs are not standardized and unanimously accepted, while the immune status of the patient regarding the antifungal agents’ activity plays the major role [20].

During the course of this postoperative infection, attempts to preserve the graft were made, while following failure of these attempts (arthroscopic debridement and antimicrobial treatment), the graft was removed and sent for histological and microbiological examination. Diagnosis was established on the removed graft and the patient commenced the appropriate antifungal treatment.

Fungal arthritis following ACLR may be devastating since most cases lead to bone loss and complex reconstructive joint surgeries or arthrodesis in relatively young patients [4,6]. Therefore, high index of suspicion and early removal of all materials (including the graft) are of utmost importance for successful outcomes.

Fungal arthritis or osteomyelitis following ACLR has a local and general mild inflammatory reaction when compared to bacterial ones. Empirical antimicrobial treatment, being usually the first step of therapeutic management, fails to control the infection. Hence, confirmed diagnosis can be delayed. Fungal infections initially cause a limited tissue reaction and progress through an indolent course. Nevertheless, they may lead to aggressive osseous destruction and necrosis [5]. A high index of suspicion is of paramount importance for early detection of such cases, while histological examination and/or detection of *Aspergillus* DNA by PCR methods may be more useful for diagnosis than cultures since cultures are more time-consuming and may vary greatly depending on several factors. Especially in recent years, *Aspergillus* PCR assays have been established for detecting early infection in high-risk patients, as well as for confirming diagnosis of established infection in real time [21]. In this case, *Aspergillus* DNA with multiplex PCR methodology in combination with the histopathological examination from the autograft specimen allowed rapid detection of the causative organism. The use of these molecular assays may lead to proper antifungal treatment in patients with invasive mold infections.

Moreover, the identification of causative *Aspergillus* spp. was made not only with the conventional techniques (e.g., macro- and microscopic examination of the isolated fungus on cultures), but also with the mass spectrometry assay. More specifically, the performance of the MALDI–TOF mass spectrometry can easily and accurately identify *Aspergillus* species, even those species that are morphologically and phylogenetically similar to each other. Hence, the use of MALDI–TOF MS-based mold analysis can drastically shorten the time to diagnosis, offering significant benefits for the patient [22].

This case reports an extremely rare infection caused by *Aspergillus fumigatus* following ACLR. Additionally, all the existing traditional and most modern diagnostic procedures were used for identification of the causative mold. Hence, this case represents an example of how diagnostic procedures supplement each other for fungal identification.

## Figures and Tables

**Table 1:** Review of all the published cases of fungal septic arthritis following ACLR. The causative fungi, definite diagnosis, as well as antifungal treatment are presented.

Authors	Year	Causative Fungus	Diagnosis	Antifungal Treatment
Burke and Zych [5]	2002	Phycomycoses	Histopathology	Amphotericin B
Muscolo et al. [6]	2009	(1). *Rhizopus* *microsporus* (2). *Rhizopus* *microsporus* (3). *Rhizopus* *microsporus* (4). *Rhizopus* *microsporus* (5). *Rhizopus* *microsporus* (6). *Candida albicans*	Cultures and histopathology	Amphotericin B
Antkowiak et al. [7]	2011	*Aspergillus flavus*	Cultures and histopathology	Voriconazole and caspofungin
Sun et al. [8]	2012	*Aspergillus* spp.	Histopathology	NR
Mirzatolooei [9]	2014	*Alternaria* spp.	Cultures	NR
Castro et al. [3]	2016	*Candida glabrata*	Cultures	Caspofungin, micafungin, voriconazole
Gamarra et al. [2]	2018	(1). *Rhizopus microsporus* (2). *Rhizopus microsporus* (3). *Rhizopus microsporus*	Histopathology, PCR	Amphotericin B

## Data Availability

Not applicable.
